# A descriptive epidemiological study of the incidence of newly diagnosed Lyme disease cases in a UK primary care cohort, 1998–2016

**DOI:** 10.1186/s12879-020-05018-2

**Published:** 2020-04-16

**Authors:** John S. P. Tulloch, Robert M. Christley, Alan D. Radford, Jenny C. Warner, Mike B. J. Beadsworth, Nick J. Beeching, Roberto Vivancos

**Affiliations:** 1 NIHR Health Protection Research Unit in Emerging and Zoonotic Infections, University of Liverpool, Liverpool, L69 3GL UK; 2 Public Health England, L3 1DS, Liverpool, UK; 3 Institute of Infection and Global Health, University of Liverpool, Liverpool, CH64 7TE UK; 4 NIHR Health Protection Research Unit in Emerging and Zoonotic Infections, Public Health England, Porton Down, SP4 0JQ UK; 5 Rare and Imported Pathogens Laboratory, Public Health England, Porton Down, SP4 0JQ UK; 6 Tropical and Infectious Disease Unit, Royal Liverpool University Hospital, Liverpool, L7 8XP UK; 7 NIHR Health Protection Research Unit in Emerging and Zoonotic Infections, Clinical Sciences, Liverpool School of Tropical Medicine, Liverpool, L3 5QA UK; 8 NIHR Health Protection Research Unit in Emerging and Zoonotic Infections, Public Health England, Liverpool, L3 1DS UK

**Keywords:** Lyme disease, Lyme borreliosis, Primary care, UK, Epidemiology, Socio-demographics, The health improvement network, THIN

## Abstract

**Background:**

Primary care is likely to see the highest number of Lyme disease patients. Despite this, there is limited published data regarding Lyme disease patients accessing primary care in the UK. We aim to describe trends in the incidence of a new diagnosis, and demographics of patients identified in a primary care electronic health database.

**Methods:**

A descriptive epidemiological study of Lyme disease coded patients in UK primary care. 3725 patients coded for Lyme disease during 1998–2016 were identified within The Health Improvement Network (THIN). Incidence rates and the demographics of cases identified were described. Poisson regression was used to analyse socio-demographic characteristics of the cases.

**Results:**

There was an increase in annual crude incidence rates, peaking in 2015 at 5.47 (95% CI 4.85–6.14) cases per 100,000 population per year. Multivariable analysis showed there were significant differences in the ages of those affected, incidence of a new diagnosis rose as deprivation levels improved, and that there was a higher incidence of cases living in rural areas compared to urban areas. There was no significant difference between sexes for the UK. Cases were significantly more likely to identify with being white compared to the national population.

**Conclusions:**

An increasing incidence of patients newly coded with Lyme disease related Read codes was identified using data from a UK national primary care database. By comparing these incidence figures with national laboratory-confirmed surveillance data, a multiplication factor of 2.35 (95%CI 1.81–2.88) can be calculated in order to estimate the annual number of cases seen in primary care. The significant socio-demographic variables associated with a Lyme disease diagnosis likely reflect a complex interplay of socio-economic issues, which needs to be further explored. Future work is needed to examine the treatment and management of patients within this database.

## Background

Lyme disease, caused by some members of the spirochaetal genospecies complex *Borrelia burgdorferi* sensu lato, has been the topic of much debate and created many headlines in the United Kingdom (UK) [1–3]. It is transmitted by the bite of an infected *Ixodes* spp. of tick, and is the most common zoonotic disease transmitted by ticks in the Northern Hemisphere [4]. It has a variety of clinical presentations, most usually including erythema migrans, flu-like symptoms, and joint and muscle pain, or more uncommonly neurological and cardiac presentations [4–7]. Current recommendations are to treat patients presenting with an erythema migrans rash with antibiotics. Laboratory diagnostic tests are recommended when erythema migrans is absent and if there is clinical suspicion and a strong supportive history of Lyme disease [7]. However, as the (National Institute for Health and Care Excellence) NICE guidelines state, ‘there is a lack of robust epidemiological data on Lyme disease in the UK’ [7]. This lack of knowledge includes incidence data in different health care settings, basic patient demographic information, and an understanding of current case management strategies by health care professionals.

As notification of clinical cases is not required, national incidence figures in the UK are based on reports of laboratory confirmed cases from the reference laboratories of Public Health England and Health Protection Scotland [7, 8]. In 2016, the national incidence was 1.95 cases per 100,000 population in England and Wales, and 3.15 cases per 100,000 in Scotland. Over the last decade, cases in England appear to be rising, whilst the incidence in Scotland is reported to be stable [7–10]. A 2016 review compared reported incidence across Western Europe and calculated a population-weighted average incidence rate of 22.05 cases per 100,000 person-years [11]. In the United States of America, a study of the incidence of clinician-diagnosed Lyme disease calculated an annual incidence of 106.6 cases per 100,000 persons [12]. These differences in incidence are likely due to a combination of differing surveillance methods and differences in true incidence. Without a comprehensive surveillance system and an internationally standardised case definition, comparisons between nations proves challenging.

Within a health care system, primary care manages the greatest number of Lyme disease patients [4, 7, 12–16]. Without understanding the potential burden for general practitioners (GPs) and the demographics of these patients, it is difficult to shape policy, deliver targeted education to the general public and clinicians, perform financial assessments, or understand case management strategies. The incidence of Lyme disease identified within primary care in the UK is poorly understood. There are two methods of recording primary care data; Read codes representing presenting symptoms or diseases, and free-text narrative. Read codes are a coded thesaurus of clinical terms that are used in primary care electronic health records in the UK and New Zealand [17]. A narrative analysis of health record free text, on a national scale, would prove ethically challenging due to difficulties in data anonymisation. On the contrary, primary care databases coded via Read codes are pseudo-anonymised and capture a large sample of the UK population. The aim of this study was to describe the incidence of a new diagnosis, and demographics of Lyme disease as recorded in primary care between 1998 and 2016 in the UK using Read code analysis.

## Methods

### Data source

Population-based primary care data from The Health Improvement Network (THIN) were used to identify patients with Lyme disease, suspected Lyme disease or Lyme disease related conditions. The design of this study was approved by the THIN Scientific Review Committee (16THIN103).

THIN collects anonymised patient data from general practices that use the VISION practice management software [18]. In 2016, this software was used by 9 % of all GP practices in England (this information is unavailable for the other UK nations; Northern Ireland, Scotland and Wales). These practices opt-in for their data to be made available in the THIN database. THIN represents 11.1 million patients with around 4.0 million annually active patients, collected from over 700 general practices in the UK. An active patient is defined as one being registered to a general practice currently supplying data to THIN, who is not dead and has not left the practice since the last data collection point. THIN has representative coverage of 6.1% of the UK population, and is representative in terms of demographics, major condition prevalence and adjusted death rates [19]. All patients and general practices are pseudo-anonymised and demographic information is available at patient level for: age, sex, ethnicity, and nation of residence. There was no available information about the geographic distribution of THIN reporting practices. However, the distribution of practice management software in English primary care in 2016 was known [20]. All these systems have high regional variability. VISION was the most geographically restricted, with coverage significantly lacking in the North and East of England. It is unknown which of the VISION practices are part of THIN, and what the geographic coverage is in other nations. We cannot conclude how geographically representative the THIN database is for the UK.

The representativeness of ethnicity data within THIN has been questioned, as the level of missingness at case-level is high. Between 2000 and 2013, 60% of THIN patient records had missing ethnicity information [21]. Ethnicity data are based upon patient-provided information categorised into the following 2011 census groups; ‘White’, ‘Mixed’, ‘Asian’, ‘Black’, and ‘Other’ [22, 23].

The remaining sociodemographic variables under assessment were Townsend scores (an indicator of material deprivation) [24], and rural urban classification. Within THIN these data are not related directly to the case but are based upon the case’s resident postcode, and then linked to 2001 census data [25]. These data are therefore not a direct measure of the case’s sociodemographics, but rather a proxy, and reflect the area that cases reside in. Townsend scores were converted, by THIN, from exact scores to quintiles of equal size. The quintile of 1 includes patients living in the lowest 20% of Townsend scores (i.e. the least deprived areas), whereas the quintile of 5 includes the highest 20% and the most deprived areas.

### Participants and statistical analysis of the data

In primary care the presenting symptoms of a patient are coded with Read codes. Currently we do not know which set of symptoms clinicians use to code the patient with ‘Lyme disease’. Our case definition was therefore restricted to Read codes specific to Lyme disease and suspect Lyme disease (Table 1).

**Table 1:** Read codes identifying Lyme disease patients in The Health Improvement Network (THIN), 1998–2016

Description	THIN Read Code	Number of patients
Lyme disease	A871000	2386
Erythema migrans	AA41.00	992
Suspected Lyme disease	1JN1.00	233
Suspected erythema migrans	1JN2.00	50
Acrodermatitis atrophicans chronica	M21y000	30
Lyme arthritis	N010A00	21
Lyme neuroborreliosis	A871100	8
Borrelial lymphocytoma	A871300	5
Lyme carditis	A871200	0
Total		3725

The ‘Suspected Lyme disease’ and ‘Suspected erythema migrans’ codes were only introduced as Read codes in 2014 [26]. Conditions with multiple aetiology, such as Bell’s palsy, were not included. This strict definition was chosen to minimise the number of false positives identified. Choosing strict case definitions will likely underestimate the number of cases and sensitivity may be lost, as cases of mixed non-specific clinical signs could be missed. These codes were used to identify patients accessing primary care between 1st January 1998 and 31st December 2016. No other exclusions were placed on the patients. The index episode was taken as the first occurrence of any one of the Read codes identified in a patient’s record. All calculations and demographic information were derived from this date. There is scant information on how to define a reinfection or relapse of Lyme disease, with no standard time period to differentiate between the two [27]. It was therefore decided that any subsequent Lyme disease Read codes associated with an identified case were not analysed. Identified cases were excluded from denominator calculations. Denominators were calculated as the total annual number of unique active patients in the THIN database. Crude annual incidence of new diagnosis rates were calculated for the whole dataset and were stratified by UK nation, month of diagnosis, and Read code. Confidence intervals of the incidence were calculated using Byar’s method. Using the Office for National Statistics (ONS) mid-year population estimates [25] and our calculated crude incidence figures, national case number estimates were calculated.

Using the cases identified within THIN and the THIN denominator population, we assessed the following variables univariably with Poisson regression; year as a linear term, sex, age, nation, Townsend quintile, and rural-urban status. Significant variables were taken forward for multivariable analysis.

Due to the poor recording of ethnicity within THIN, the complete electronic health record of each identified case was read to confirm ethnicity status, rather than constructing a Read code search. It was not feasible to read and confirm the denominator population and therefore incidence could not be calculated. Instead, proportions of ethnicity classification for cases were calculated and compared to the ONS national population ethnicity data, using a Chi-squared test.

A ratio between the incidence of new diagnosed cases in primary care and the incidence of laboratory-confirmed cases was created. National laboratory data for the UK is available between 2007 and 2016 and released in the UK government’s annual Zoonoses report [7]. An annual ratio was calculated by dividing the crude annual incidence of new diagnosis for the THIN dataset by the annual incidence published in the Zoonoses report. The mean annual ratio, with associated confidence intervals, was calculated. All statistical analyses were carried out using R language (version 3.2.0) (R Core Team 2015), and results were deemed significant where *p* < 0.05.

## Results

In total 3725 unique patients were identified with a Read code for Lyme, suspected Lyme disease, or related conditions Read code between 1st January 1998 and 31st December 2016 (Table 1). The most frequently used Read codes (‘Lyme disease’ and ‘Erythema migrans’) represented 89.1% (*n* = 3318) of all Read codes identified with Lyme disease. The suspected Lyme disease codes only represented 7.6% (*n* = 283) of all codes.

**Fig. 1:**
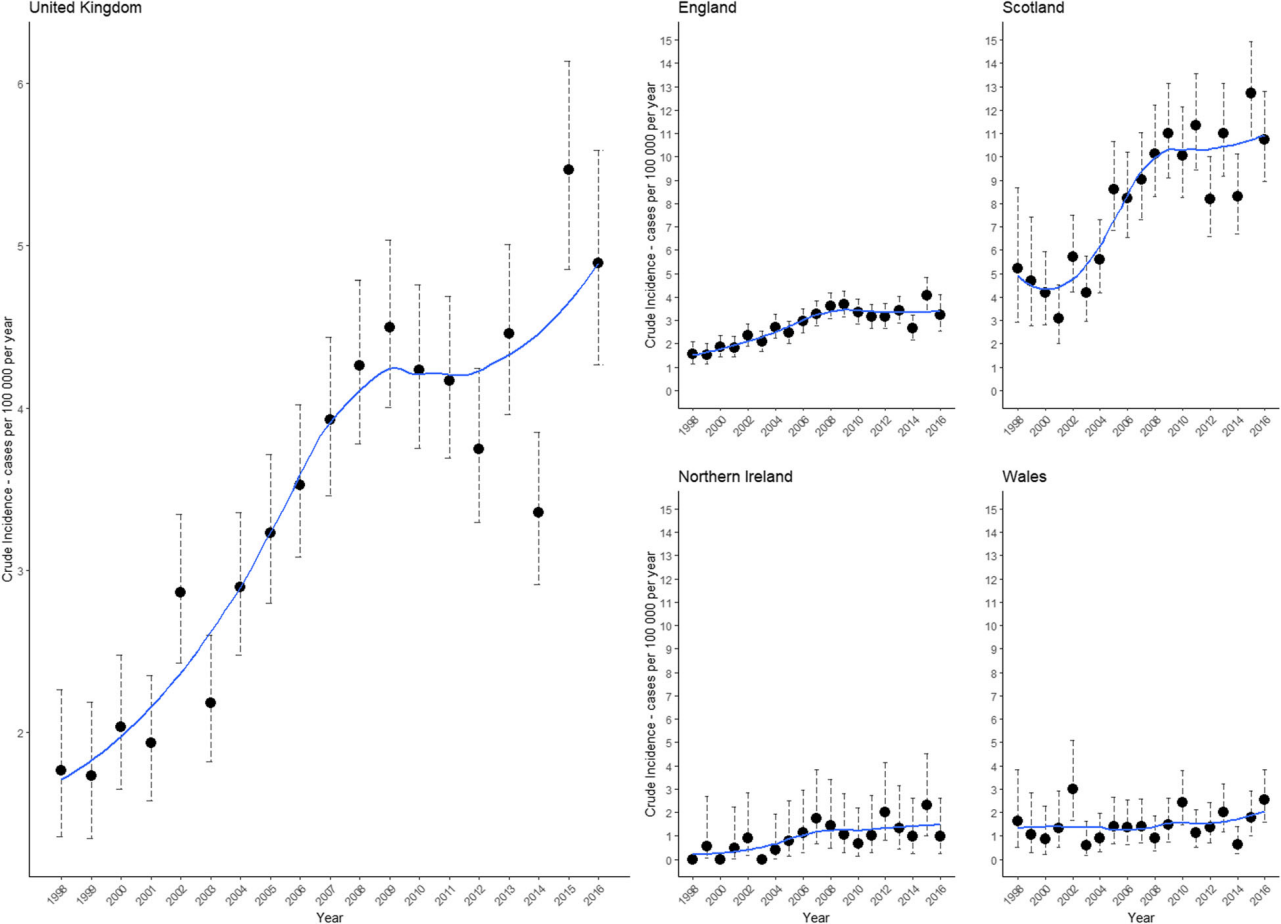
Crude incidence of UK Lyme disease cases in The Health Improvement Network (THIN), 1998–2016. Dotted lines represent 95% confidence intervals. Smoothed lines of best fit were calculated using the LOESS method

There was an increase in the crude incidence of a new diagnosis of Lyme disease in UK primary care between 1998, 1.77 (95% CI 1.35–2.26) cases per 100,000, and 2016, 4.89 (95% CI 4.26–5.59) cases per 100,000 (Fig. 1, Table 2).

**Table 2:** Incidence of a new diagnosis of Lyme disease in UK primary care and estimated number of cases

Year	UK incidence	UK cases	England incidence	England cases	N.I. incidence	N.I. cases
1998	1.77 (1.35–2.26)	1035 (789–1321)	1.56 (1.14–2.09)	762 (557–1020)	0 (0–1.62)	0 (0–27)
1999	1.73 (1.35–2.19)	1015 (792–1285)	1.52 (1.13–2.01)	745 (554–986)	0.58 (0.05–2.68)	10 (1–45)
2000	2.03 (1.65–2.48)	1195 (972–1460)	1.85 (1.43–2.36)	911 (704–1162)	0 (0–1.22)	0 (0–21)
2001	1.93 (1.58–2.35)	1141 (934–1389)	1.83 (1.43–2.32)	905 (707–1147)	0.48 (0.04–2.22)	8 (1–37)
2002	2.86 (2.43–3.35)	1698 (1443–1989)	2.34 (1.89–2.87)	1162 (939–1426)	0.89 (0.18–2.86)	15 (3–49)
2003	2.18 (1.82–2.60)	1300 (1085–1551)	2.08 (1.67–2.57)	1038 (834–1283)	0 (0–1.08)	0 (0–18)
2004	2.89 (2.48–3.36)	1733 (1487–2014)	2.72 (2.25–3.26)	1365 (1129–1636)	0.42 (0.04–1.94)	7 (1–33)
2005	3.23 (2.80–3.71)	1951 (1692–2241)	2.46 (2.02–2.97)	1245 (1022–1503)	0.78 (0.15–2.48)	13 (3–43)
2006	3.53 (3.08–4.02)	2147 (1873–2445)	2.96 (2.49–3.51)	1509 (1269–1789)	1.11 (0.31–2.97)	19 (5–52)
2007	3.92 (3.46–4.43)	2404 (2122–2716)	3.29 (2.79–3.86)	1690 (1434–1983)	1.74 (0.66–3.82)	31 (12–67)
2008	4.26 (3.78–4.79)	2634 (2337–2961)	3.60 (3.08–4.19)	1865 (1596–2171)	1.43 (0.48–3.40)	25 (9–60)
2009	4.50 (4.00–5.03)	2802 (2490–3132)	3.68 (3.15–4.27)	1921 (1644–2229)	1.05 (0.29–2.81)	19 (5–50)
2010	4.23 (3.75–4.76)	2655 (2353–2987)	3.36 (2.84–3.94)	1769 (1495–2074)	0.69 (0.14–2.22)	12 (3–40)
2011	4.17 (3.69–4.69)	2639 (2335–2968)	3.15 (2.66–3.71)	1673 (1413–1970)	1.02 (0.28–2.73)	19 (5–50)
2012	3.75 (3.29–4.24)	2389 (2096–2701)	3.18 (2.68–3.74)	1701 (1434–2001)	2.02 (0.84–4.16)	37 (15–76)
2013	4.46 (3.96–5.01)	2859 (2539–3212)	3.42 (2.89–4.02)	1842 (1557–2165)	1.33 (0.44–3.16)	24 (8–58)
2014	3.36 (2.91–3.85)	2170 (1880–2487)	2.66 (2.17–3.22)	1445 (1179–1749)	0.98 (0.27–2.63)	18 (5–48)
2015	5.47 (4.85–6.14)	3562 (3158–3998)	4.06 (3.38–4.84)	2224 (1852–2652)	2.30 (1.03–4.52)	43 (19–84)
2016	4.89 (4.26–5.59)	3210 (2797–3670)	3.25 (2.55–4.10)	1796 (1409–2266)	0.98 (0.27–2.60)	18 (5–48)
Year			Scotland incidence	Scotland cases	Wales incidence	Wales cases
1998			5.21 (2.92–8.66)	265 (148–440)	1.61 (0.53–3.82)	47 (15–111)
1999			4.68 (2.78–7.42)	237 (141–376)	1.07 (0.30–2.85)	31 (9–83)
2000			4.17 (2.83–5.93)	211 (143–300)	0.85 (0.23–2.26)	25 (7–66)
2001			3.09 (2.23–4.51)	156 (113–228)	1.33 (0.50–2.91)	39 (15–85)
2002			5.70 (4.24–7.51)	289 (215–381)	3.01 (1.65–5.10)	88 (48–149)
2003			4.17 (2.96–5.73)	211 (150–290)	0.61 (0.17–1.64)	18 (5–48)
2004			5.59 (4.20–7.31)	284 (214–372)	0.90 (0.34–1.97)	27 (10–58)
2005			8.62 (6.87–10.68)	440 (351–546)	1.40 (0.66–2.64)	42 (20–78)
2006			8.22 (6.54–10.22)	422 (336–525)	1.34 (0.63–2.53)	40 (19–76)
2007			9.03 (7.30–11.06)	467 (377–572)	1.42 (0.70–2.58)	43 (21–78)
2008			10.13 (8.31–12.23)	527 (432–636)	0.91 (0.38–1.87)	28 (11–57)
2009			11.00 (9.11–13.16)	576 (477–689)	1.47 (0.76–2.62)	45 (23–80)
2010			10.06 (8.27–12.13)	529 (435–638)	2.43 (1.47–3.80)	74 (45–116)
2011			11.37 (9.46–13.55)	603 (501–718)	1.13 (0.53–2.13)	35 (16–65)
2012			8.18 (6.59–10.03)	435 (350–533)	1.37 (0.71–2.44)	42 (22–75)
2013			11.02 (9.17–13.13)	587 (489–700)	2.01 (1.17–3.23)	62 (36–100)
2014			8.29 (6.72–10.13)	443 (359–542)	0.65 (0.24–1.41)	20 (7–44)
2015			12.71 (10.73–14.94)	683 (577–803)	1.79 (1.03–2.92)	55 (32–90)
2016			10.74 (8.94–12.80)	580 (483–692)	2.54 (1.60–3.85)	79 (50–120)

NI = Northern Ireland

**Fig. 2:**
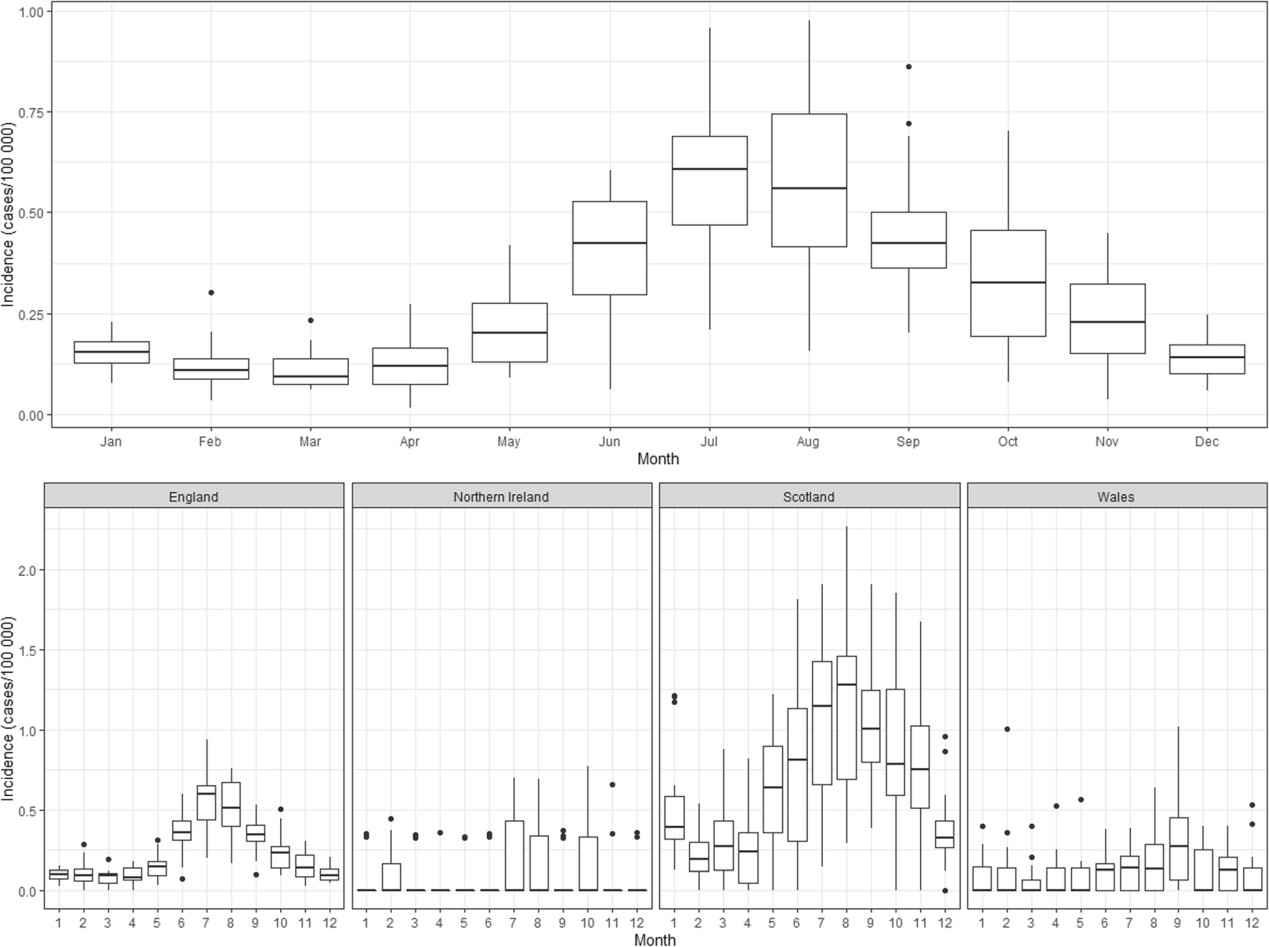
Monthly incidence of Lyme disease in The Health Improvement Network (THIN), 1998–2016

This rise was seen in all nations except Wales. Across the UK, cases displayed a seasonal pattern, with the highest incidence of a new diagnosis in the summer, peaking in July and August (Fig. 2). This seasonality was seen in England and Scotland with incidence peaking in July and August respectively. In Northern Ireland and Wales no obvious trends were seen.

**Fig. 3:**
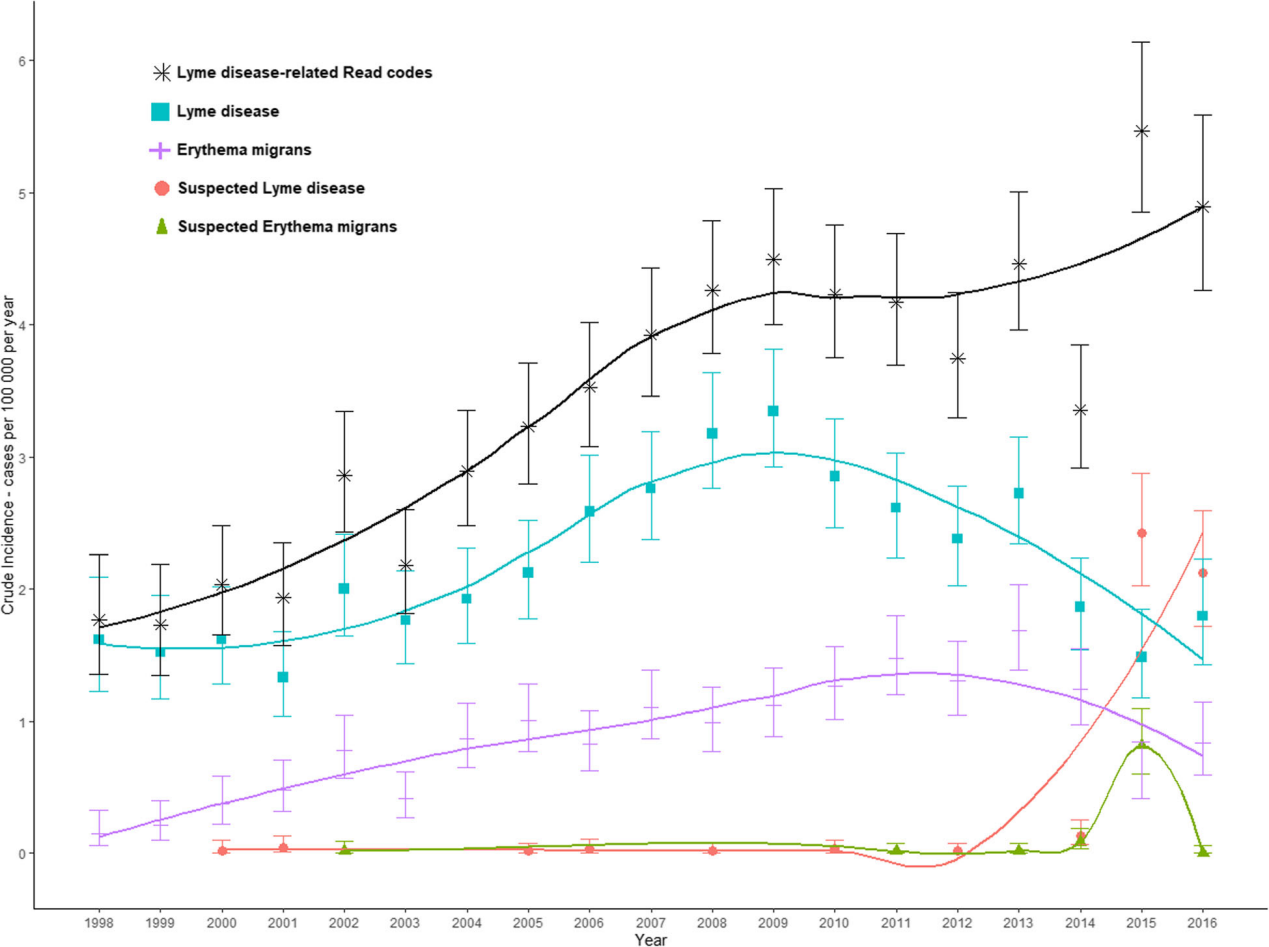
Crude incidence of Lyme disease Read codes in The Health Improvement Network (THIN), 1998–2016. Dotted lines represent 95% confidence intervals. Smoothed lines of best fit were calculated using the LOESS method. Only the four most prevalent Lyme disease Read codes are displayed

The ‘Lyme disease code’ rose until a peak in 2009 before steadily declining (Fig. 3). ‘Erythema migrans’ had a lower incidence and peaked in 2011 before declining. Both ‘Suspected Lyme disease’ and ‘Suspected erythema migrans’ showed a sharp increase in incidence in 2015. In 2016 ‘Suspected Lyme disease’ was the most prevalent Read code in use.

All variables examined were statistically significant in the univariable Poisson analysis and were taken forward for multivariable analysis (Table 3).

**Table 3:** Univariable and multivariable Poisson regression analyse for Lyme disease incidence of a new diagnosis in the UK

	Univariable Analysis		Multivariable Analysis Model 1		Multivariable Analysis Model 2	
Independent Variable	IRR (95% CI)	*p*-value	IRR (95% CI)	*p*-value	IRR (95% CI)	*p*-value
Year (linear)	1.05 (1.05–1.06)	< 0.001	1.05 (1.04–1.06)	< 0.001	1.04 (1.03–1.05)	< 0.001
Sex						
Male	1		1		1	
Female	1.08 (1.01–1.15)	0.02	1.06 (0.99–1.13)	0.08	1.20 (1.10–1.30)	< 0.001
Age (Years)						
< 1	0.04 (0.002–0.18)	0.001	0.04 (0.003–0.20)	0.002	0.07 (0.004–0.31)	0.008
1–5	0.55 (0.42–0.69)	< 0.001	0.52 (0.40–0.67)	< 0.001	0.65 (0.48–0.88)	0.006
6–10	0.76 (0.63–0.91)	< 0.001	0.72 (0.59–0.87)	< 0.001	0.95 (0.75–1.20)	0.67
11–15	0.66 (0.55–0.80)	< 0.001	0.67 (0.55–0.81)	< 0.001	0.88 (0.70–1.11)	0.29
16–20	0.63 (0.52–0.75)	< 0.001	0.64 (0.53–0.78)	< 0.001	0.87 (0.69–1.10)	0.25
21–25	0.64 (0.54–0.77)	< 0.001	0.68 (0.56–0.81)	< 0.001	0.85 (0.68–1.07)	0.18
26–30	0.78 (0.66–0.92)	0.004	0.81 (0.68–0.96)	0.02	0.96 (0.77–1.20)	0.74
31–35	0.80 (0.68–0.94)	0.006	0.85 (0.72–1.01)	0.06	0.86 (0.68–1.07)	0.17
36–40	0.82 (0.70–0.96)	0.012	0.83 (0.70–0.98)	0.02	0.89 (0.72–1.11)	0.30
41–45	1		1		1	1
46–50	0.98 (0.85–1.14)	0.83	0.97 (0.83–1.13)	0.72	0.94 (0.77–1.17)	0.60
51–55	0.98 (0.84–1.14)	0.80	0.92 (0.79–1.08)	0.33	0.92 (0.74–1.14)	0.45
56–60	1.27 (1.09–1.47)	0.002	1.23 (1.06–1.44)	0.007	1.25 (1.02–1.53)	0.03
61–65	1.25 (1.07–1.45)	0.004	1.19 (1.02–1.39)	0.03	1.02 (0.82–1.26)	0.89
66–70	1.01 (0.86–1.20)	0.86	0.99 (0.83–1.17)	0.89	0.98 (0.77–1.23)	0.84
71–75	0.89 (0.73–1.07)	0.21	0.89 (0.73–1.07)	0.21	0.85 (0.66–1.10)	0.23
76–80	0.63 (0.50–0.79)	< 0.001	0.66 (0.52–0.83)	< 0.001	0.77 (0.57–1.02)	0.07
81–85	0.43 (0.32–0.57)	< 0.001	0.46 (0.33–0.61)	< 0.001	0.50 (0.34–0.73)	< 0.001
> 85	0.13 (0.08–0.20)	< 0.001	0.14 (0.09–0.23)	< 0.001	0.17 (0.09–0.29)	< 0.001
Nation						
England	1		1		N/A	
Northern Ireland	0.36 (0.27–0.47)	< 0.001	0.36 (0.26–0.48)	< 0.001	N/A	
Scotland	3.01 (2.82–3.22)	< 0.001	3.16 (2.95–4.76)	< 0.001	N/A	
Wales	0.52 (0.44–0.61)	< 0.001	0.56 (0.47–0.66)	< 0.001	N/A	
Townsend						
1	1		1		1	
2	1.29 (1.18–1.41)	< 0.001	1.12 (1.02–1.22)	0.01	0.88 (0.79–0.99)	0.03
3	0.94 (0.85–1.03)	0.21	0.84 (0.76–0.92)	< 0.001	0.66 (0.59–0.75)	< 0.001
4	0.64 (0.58–0.72)	< 0.001	0.54 (0.49–0.61)	< 0.001	0.55 (0.48–0.64)	< 0.001
5	0.41 (0.35–0.48)	< 0.001	0.32 (0.28–0.37)	< 0.001	0.43 (0.35–0.52)	< 0.001
Rural Urban						
Urban	1				1	
Rural	1.96 (1.78–2.15)	< 0.001	N/A	N/A	1.71 (1.56–1.89)	< 0.001

*IRR* Incidence rate ratio, *CI* Confidence Interval

The age band 41–45 years was chosen as the reference for age analysis as this group contained the mean age for the dataset (mean = 42.9, 95% CI 42.3–43.6). Information relating to Townsend score was available for 93.6% (*n* = 3487) of cases. Rural urban classifications were only available for English and Welsh cases, as Scottish and Northern Ireland authorities do not record this measure. Therefore, two multivariable models were created. Model 1 excluded the rural urban classification, whilst model 2 excluded nation and maintained the rural urban classification.

Univariable analysis showed a significant increase in the incidence of a new diagnosis with each year. There was a significantly higher incidence of new diagnosis in women compared to men. Most age bands had a statistically significantly lower incidence to the reference range. Adults between 56 and 65 had a significantly higher incidence. There was no significant difference in incidence between the following age groups; 46–50, 51–55, 66–70 and 71–75. Scotland had a statistically significant higher incidence compared to England, whilst Wales and Northern Ireland had a significantly lower incidence. The incidence rate ratio was significantly higher in the second quintile, the same in the third and then declined as Townsend quintile increased. There was a statistically significantly higher incidence in patients residing in rural areas compared to urban areas.

Multivariable analysis showed the same patterns in significance for year and nation (Table 3, model 1). There was no longer a significant difference between sexes. The only age band to change was 31–35, which was no longer significant. Townsend quintiles showed a significant decrease in incidence rate ratio as Townsend quintile increased, apart from quintile 2 which was significantly higher than 1.

Model 2 excluded nation from analysis but included rural urban classification, thus essentially representing a model of just English and Welsh cases. Incidence of a new diagnosis significantly increased with each year. Women had a significantly higher incidence than men. The age band variable changed the most compared to univariable analysis. All ages were no longer statistically different to the reference age except, < 1, 1–5, 81–85,> 85 which were significantly lower, and 56–60 which was significantly higher. The Townsend quintiles showed a clear trend with incidence significantly declining as quintile increased. Incidence of new diagnosis was significantly higher in rural areas compared to urban areas.

There was a high degree of missing data for ethnicity, with only 35.1% (*n* = 1306) of cases providing information. Of these, 73.5% (*n* = 960), had an ethnicity description that matched the ethnicity categories defined in the UK 2011 census [23]; the remaining 346 all identified with being ‘British/Mixed British’. There was a significant difference in ethnic diversity (*p* < 0.01), with a higher percentage of the Lyme disease coded THIN patients (96%) identifying with being white compared to the national population (87%).

The mean annual ratio between THIN crude incidence figures and national laboratory-confirmed incidence figures was 2.35 (95% CI 1.81–2.88). The ratio ranged from 1.91 in 2012 to 2.82 in 2015.

## Discussion

This study describes the incidence of a new diagnosis, and demographics of Lyme disease coded patients using primary care data in the UK, fulfilling one of the key research needs identified by the NICE guidelines [7]. There has been an increase in the annual incidence of newly coded Lyme disease patients in UK primary care between 1998 and 2016. Incidence varied between nations, with Scotland experiencing the highest incidence of disease. There was a higher incidence of Lyme disease coded THIN patients living in rural areas and within areas of lower deprivation.

A UK study estimating Lyme disease incidence in primary care showed a higher incidence, with 12.1 cases per 100,000 in 2012 [28]. This was about three times our estimate for 2012 (Table 2). Our study used a more specific case definition, in line with the NICE clinical guidelines [7], and is likely to provide a conservative estimate. Therefore, it is not possible to directly compare our results with those of the above paper as different case definitions were used and no stratification of results were provided. European studies using similar data from primary care sentinel practices have described a large range in incidence [14, 29, 30], from 42 cases per 100,000 per year in France [13], to 148 per 100,000 per year in Norway [15]. The UK had a much lower incidence rate across the study period; 4.23 (95% CI 4.09–4.34) cases per 100,000 person-year. The UK had its peak crude incidence of new diagnosis in 2015, 5.47 (95% CI 4.85–6.14) per 100,000 population. The annual incidence significantly varied between nations; Scotland peaked in 2015 with an incidence of 12.71 (95% CI 10.73–14.94), England in 2015 with 4.06 (95% CI 3.38–4.84), Northern Ireland in 2015 with 2.30 (95% CI 1.03–4.52), and Wales in 2016 with 2.54 (95% CI 1.60–3.85). Even in Scotland, the incidence of Lyme disease is lower than in most areas of continental Europe. The reasons for this are likely to be multiple and need to be further explored. They may include; a lower prevalence of *Ixodes spp* of ticks, a lower prevalence of *Borrelia spp* carriage by ticks (4.2% in southern England [31], 0–8.2% in northern England [32], and 10.2% in Scotland [33], compared to 13.6% across Europe [34]), and different levels of exposure of the general populace to ticks, possibly due to differences in occupational and/or recreational dispositions. One possible explanation is lower awareness about Lyme disease in the general population and primary care in the UK, compared to the rest of Europe. This would result in fewer presentations to primary care, the potential for mis-diagnosis and a resultant underreporting of cases.

These differences in incidence of new diagnosis between nations are notable and likely to be multifactorial. Scottish GPs may be more confident in diagnosing a case of Lyme disease, due to the higher prevalence of Lyme disease compared to England and Wales [7], and so manage more patients within primary care without submitting samples for serological testing. English and Welsh GPs could be more reluctant to diagnose and treat Lyme disease cases and may refer cases to secondary care sooner than their Scottish equivalents. There may be differences in patient access to primary care or differences in health-seeking behaviour between the different nations, dependent on differing clinical presentations. Further analysis of the THIN database may provide information about case referrals, and differences in case presentation and management. However, the exploration of differences in GP recording or patient behaviour would best conducted through qualitative research.

The incidence figures are notable higher than those reported in current surveillance figures based on laboratory confirmed cases [10]. The laboratory confirmed incidence of Lyme disease in England and Wales in 2016 was 1.95 cases per 100,000 (95%CI 1.84–2.06) [7–9], whilst that identified in THIN was 3.06 (95%CI 2.47–3.75). The laboratory confirmed incidence of Lyme disease in Scotland in 2016 was 3.15 cases per 100,000 (95%CI 2.70–3.65) [7–9], in THIN it was 10.74 (95%CI 8.94–12.80). The laboratory confirmed incidence of Lyme disease in Northern Ireland in 2016 was 0.21 cases per 100,000 (95%CI 0.07–0.52) [7–9], in THIN it was 0.98 (95%CI 0.27–2.60). The large non-overlapping differences suggest that the incidence described in primary care data, for each nation of the UK, was significantly larger than that described by official laboratory confirmed cases. The exception was Northern Ireland, which may be due to the sparsity of Northern Irish cases in the THIN dataset.

The mean annual ratio between primary care and laboratory confirmed incidence Figs. (2.35 (95%CI 1.81–2.88)) suggest that for every laboratory-confirmed case there are about two cases potentially identified within UK primary care practices. This was to be expected as not all cases of Lyme disease (in particular those with erythema migrans) require confirmatory diagnostic laboratory tests. This ratio could be used as a multiplication factor to estimate the number of annual cases seen in primary care based on laboratory confirmed cases.

The rise in the annual incidence of a new diagnosis of Lyme disease, and the differences in incidence with the laboratory datasets, could be a result of a real increase in disease, an increasing awareness of the disease in the general public, a change in general practitioners’ behaviour resulting in the submission of fewer diagnostic samples, or a combination of the above. Further work is needed to understand how general practitioners diagnose and manage Lyme disease cases. Wales is the only nation that does not have an obvious increase in cases, which may be due to, at least in part, a low number of cases (*n* = 165) and registered THIN practices in Wales. The peak number of cases we observed in summer months is consistent with other studies [7, 10, 35–37]. This peak occurs slightly earlier in England than in Scotland. This is likely due to latitudinal, climatic and ecological differences between the two nations impacting on, the emergence and peak numbers of nymphal ticks [38]. The low case numbers in Wales and Northern Ireland (*n* = 50) likely explain the lack of an obvious seasonal trend.

Analysis of Lyme disease patient demographics have shown predominance in both sexes in a variety health care settings in the UK [7, 9, 10, 36, 37, 39]. In comparison to other national primary care datasets, Switzerland and France have no statistical difference between sexes, but numerically more women [13, 29]. Finland and Norway have significantly more women [14, 15]. The results from THIN indicate no difference in the incidence between sexes at a national level. However, local differences may exist relating to differences in tick exposure or presentation to health services [40]. This was exemplified by the second model, representing England and Wales, that had a higher incidence in women.

There is building evidence that areas with higher Lyme disease incidence are likely to be less deprived [10, 37, 41, 42]. The current analysis was able to show that socio-economic and rural-urban status were significantly and independently associated with Lyme disease incidence. There is obviously a complex interplay between ethnicity, socio-economic status and place of residence of a case, probably related with either outdoor employment or leisure activities. The results add to previous hypotheses that use and access to the countryside is a driver of Lyme disease risk.

In England 45% of all outdoor visits were to the countryside, 68% of these were within two miles from their starting point (usually a home address), and that people were less likely to visit if they were from a BAME (Black, Asian, minority ethnic) background, or from a ‘DE’ social group (i.e. semi-skilled and unskilled occupations, unemployed and lowest grade occupations) [43]. In Scotland, 50% of outdoor visits were taken in the countryside, the average distance travelled from home being 4.8 miles, and that people were less likely to visit if they lived in the 15% most deprived areas, and were of ‘DE’ social grade; no difference in regards to ethnicity was identified [44]. All ethnic minority groups are more likely to live in areas of higher deprivation compared to the white population, and there is a lack of ethnic diversity in wealthy areas [45]. Taking this into consideration, we believe that members of the general population who live in areas of low deprivation, predominantly rural locations [46, 47], are more likely to identify with a white ethnicity, and due to their residential location have greater and closer access to the countryside. This increased potential access to the countryside enables increased risk of a tick bite and therefore subsequent risk of developing Lyme disease. The lack of representation of non-white ethnicity patients may also be due to inadequate healthcare access, lack of Lyme disease awareness, or simply that erythema migrans rashes are harder to identify on non-white skin colour [48, 49]. The latter assumption would not hold true with other clinical presentation, and it is recommended that ethnicity should be explored in relation to clinical presentation prevalence. The ethnicity data has a high degree of missingness, 74%, more so than prior analyses, 60% [21]. Its representativeness must therefore be questioned; our data only provides a general indicator of the true situation.

With this large scale work we provide UK specific baseline data that is greatly need for further epidemiological research on Lyme disease, and have fulfilled one of the NICE guidelines identified research needs [7]. We have highlighted new insights into the demographics of Lyme disease patients in primary care. THIN has been shown to be representative of the UK population and as such the results are likely to be representative of the Lyme disease cases seen in primary care. The majority of research investigating conditions within a primary care database also try to validate the Read codes investigated. This is usually via the result of a diagnostic test or a questionnaire of general practitioners [50, 51]. Validation of Read codes relating to Lyme disease therefore prove a challenge, as if there is an uncomplicated clinical presentation the clinician is recommended to prescribe antibiotics without performing subsequent diagnostic tests [7]. Therefore, matching a Read code case with a positive test result may be a fruitless exercise. Instead, validation through a GP questionnaire would be recommended. In the majority of cases there will be no confirmatory diagnostics, so GPs would have to confirm a case by remembering the exact consultation, as the information collected by THIN does not substantially differ from what is in the practices’ clinical records. Hence, there would be scope for considerable error. Methodology for validating conditions with broad clinical presentations needs to be explored, but this was beyond the scope of this study.

### Limitations

One of the largest limitations of this study is the absence of knowledge about GP coding practices and changes in their coding behaviour. Further work is required to better understand coding practices and how they may vary. The Read codes used by clinicians were consistent until 2010, with the majority being ‘Lyme disease’ and ‘Erythema migrans’, at which point the use of these terms started to decline. A year after the introduction of the ‘suspected’ case codes in 2014, the ‘suspected’ codes were already more prevalent in use than ‘Lyme disease’ and ‘Erythema migrans’. The reasons for the changes in GP coding behaviour, potentially indicated by changes in code incidence, are unknown; the change may be due to the increasingly politicised landscape of Lyme disease and the debate around ‘chronic Lyme disease’ [2, 52]. We need to know what symptoms are identified to code a patient with ‘Lyme disease’; this could be only an erythema migrans rash or another presentation described by NICE [7]. Qualitative research around general practitioners’ recognition and coding behaviour regarding Lyme disease would help answer these questions. Only 25.8% (*n* = 960) of the study population had information that could be analysed around ethnicity. We assumed that the trends seen in this subset of patients is representative of the THIN population as a whole; further work is needed to verify this. Finally, the geographical resolution of THIN only allows us to carry out analysis to the level of the constituent nations of the UK, so analysis of the spatial distribution of incidence with this dataset is not possible. Previous research in the UK has shown clear clustering of cases both from laboratory confirmed cases [7, 9, 36], and hospital admissions [37, 39]. The largest number of identified cases will be in primary care, because not all cases require diagnostics or hospital admissions. Therefore, without greater resolution, we cannot see whether the observed hotspots of disease in laboratory surveillance systems are reflected in primary care activity.

The multivariable Poisson regression must be treated with a degree of caution as some of the data falls into ecological fallacy. Both Townsend quintile and rural-urban status are based upon information regarding the area in which the patient resides rather than about the individual patient. In no UK health datasets are these variables directly attributable to the patient rather than a geographic area. As these are important variables to explore the authors felt justified in analysing the data using this methodology. The strong and significant associations suggest that this approach was justified. However ecological fallacy must be acknowledged until the time that these variables can be explored at patient level on a national scale.

The NICE guidelines highlighted the ‘lack of robust epidemiological data’ on Lyme disease in the UK, and called for research in this area [7]. This research provides a description of the demographics and incidence of a representative UK primary care population. This work will help ensure the appropriate public health prioritisation of Lyme disease in the UK, however, many basic epidemiological questions remain unanswered. These mainly revolve around person-tick interaction and include; the *Borrelia* spp. seroprevalence of the UK population, the total exposure of tick bites to the UK population, and the risk of contracting Lyme disease after a tick bite in the UK. These final two points could be explored using the THIN dataset.

## Conclusions

Our data provides the primary care practitioners with basic sociodemographic information about the type of patient who is more likely to present with Lyme disease. This information can be used to raise awareness of increasing Lyme disease presentations in primary case and their seasonality in the UK. This information is critical to their diagnostic clinical decision making and ensures that their clinical suspicion of Lyme disease is increased in suitable situations. This research, alongside the NICE guidelines [7], will raise Lyme disease awareness amongst primary care clinicians and thus ensure that Lyme disease is aptly placed on their differential diagnosis list. Patients are therefore less likely to be misdiagnosed and will be managed more appropriately. This will only enhance Lyme disease patient care.

A multiplication factor was identified which could be utilised to estimate the number of Lyme disease cases seen in primary care. Comparing Lyme disease presentations in primary care with incidence in laboratory surveillance systems can highlight areas where differences exist regarding awareness, reporting, and management of Lyme disease. These differences would require further investigation. Future research using Lyme disease coded patients within THIN, will investigate concurrent symptoms, and treatment and referral choices as part of case management plans. This study provides a platform to describe patient management in the UK primary care setting and enables ongoing epidemiological analysis of Lyme disease.

## Data Availability

The data that support the findings of this study are available from THIN, but restrictions apply to the availability of these data, which were used under license for the current study, and so are not publicly available. Data are however available from the authors upon reasonable request and with permission of THIN.

## Abbreviations

BAME: Black, Asian, and minority ethnic
GPs: General practitioners
NICE: National Institute for Health and Care Excellence
NI: Northern Ireland
ONS: Office for National Statistics
THIN: The Health Improvement Network
UK: United Kingdom
